# Change competence: An integrative literature review

**DOI:** 10.3233/WOR-230633

**Published:** 2024-10-07

**Authors:** Cathrine Reineholm, Daniel Lundqvist, Andreas Wallo

**Affiliations:** Department of Behavioural Sciences and Learning, Linköping University, Linköping, Sweden

**Keywords:** Organizational change, change management, organizational innovation, workplace, learning, review

## Abstract

**BACKGROUND:**

Organizations are in a state of continual evolution, driven by the relentless shifts in their external environments. Numerous theories have been proposed to understand the essential skills and capabilities for successful organizational change. Yet, there remains a gap in capturing a holistic view necessary to fully comprehend the dynamics of competence in today’s rapidly changing landscape.

**OBJECTIVE:**

This research aims to explore and consolidate the concept of ‘competence’ in the context of organizational change processes.

**METHODS:**

Employing an integrative literature review approach, a total of 3,230 studies were screened. Out of these, 32 studies were selected based on strict relevance and quality criteria, providing a robust foundation for the analysis.

**RESULTS:**

The findings reveal a multi-layered nature of organizational change, highlighting that the nature and prerequisites of change vary significantly across different organizational levels. By applying a competence lens, we discern how required competence during change are not uniform but rather vary depending on whether they are applied in an operational or strategic context. This demonstrates a nuanced, level-dependent variability in change competence across the organizational hierarchy.

**CONCLUSION:**

We conceptualize ‘change competence’ as a dual-faceted construct. It encompasses both the capacity to leverage existing organizational competence and the adeptness to develop new competence, thereby meeting the evolving demands imposed by both internal and external drivers of change. This comprehensive understanding paves the way for more effective strategies in managing organizational change.

## 1 Introduction

Organizational change is often described as necessary for organizations to survive in a dynamic environment with different, often external, threats. These challenges force organizations to adapt and align to new organizational goals and procedures, as well as new work content or work tasks for the employees [1, 2]. In recent years, organizations in all sectors have faced several new challenges, e.g. digitalization and artificial intelligence, remote work and flexible workplaces [3–5], sustainability and green transition. However, the demand for organizations to be transformable is no recent phenomenon. Rather, grasping how and why organizations change has long been a central quest of researchers in various academic disciplines [6]. Change can be seen as an ever-present element affecting all organisations to varying degrees [7]. It has been argued that organizational change is, in fact, the new normal state [8]. Organizational structures, work content, and production systems are constantly evolving as a response to continuous changes in the external context [9]. Implementing change is, however, a challenge as all individuals in the organization need to cooperate [1].

As a result, disciplines such as change management (CM), organizational development (OD), and human resource development (HRD) have witnessed numerous influential contributors striving to explain the nature of organizational change [9]. Research within these disciplines underscores the necessity of understanding various change models, including Lewin’s Change Management Model, the McKinsey 7S Model, and Kotter’s 8-Step Change Model, to navigate change processes effectively.

According to Santos et al. [10], strategic CM is required to succeed in implementing change. The role of HRD in ensuring that people within an organization possess and develop the requisite skills to support change has been a prominent focus, particularly in training and development. Although the detailed focus on individual skills and capacities is crucial for practical applications, it may prevent a comprehensive understanding of competence at all organizational levels during change. Admittedly, there are adjacent concepts, such as “dynamic capabilities” [11], “absorptive capacity” [12], “corporate competencies” [13], and ”organizational change capacity” [14]. Still, these do not capture the holistic perspective that we believe is needed to understand competence requirements in today’s rapid change pace. Competence during a change may differ depending on work tasks, work experience, work assignment or profession, and hierarchical level, as well as if the change is initiated top-down or bottom-up. These concepts should, instead, be seen as essential parts of an overarching competence concept during organizational change.

As such, through an integrative literature review, this paper aims to explore and consolidate the concept of ‘competence’ in the context of organizational change processes. Two research questions have been formulated: What does competence imply during a change process? What does competence at different organizational levels entail?

### 1.1 Organizational change

Organizational change refers to the process through which an organization transitions to adjust or improve its operations, structures, processes, strategies, or culture. This change can be driven by internal factors such as leadership shifts, operational improvements, or cultural transformations or by external factors like technological advancements, market trends, economic shifts, or regulatory changes [6, 7, 15].

The concept of change has been given many different understandings depending on theoretical perspectives [6, 16]. Change can be unprecedented, ambiguous and hard to grasp, but it can also be planned, foreseen and managed [8]. The focus of this review is on planned changes, which refer to intentional activities that aim to change all or parts of an organization from its current state to a desired future state [15]. These planned activities can include both internal and external organizational conditions. This review concentrates explicitly on planned changes because of the previously mentioned rapid transformations associated with the green transition, the implementation of hybrid work arrangements, and the introduction of generative Artificial Intelligence for public use. Thus, the review focuses on proactive and strategic change management approaches to effectively navigate and capitalize on these rapidly evolving domains rather than on emergent changes that arise spontaneously and may not align with the deliberate goals of organizations in these contexts.

### 1.2 Competence

The concept of competence holds different meanings and has been challenging to define, often due to inconsistent usage [17] or conceptual ambiguities [18]. According to Lo et al. [19], the literature has traditionally leaned towards either a behavioural or personal attribute approach when discussing competence. Both approaches focus on identifying sets of individual competences that differentiate successful from less successful performers. This leads to a universalist perspective that competence can be generic or universally applicable to multiple occupations, irrespective of context. While there is merit to both approaches, the lack of contextual considerations paved the way for a third approach, the interpretative one [20]. In the interpretative approach, sometimes referred to as the situational approach, competence is understood as knowledge in action and understanding of work or practice [21, 22].

In this review, we argue that there is merit to applying a broader perspective and the interpretative view, where competence is not limited to individuals but extends to groups and organizations. It is a potential capacity that may not be inherently accommodated in planned change efforts. This capacity comprises five key components: perceptual motor skills, such as dexterity; cognitive factors, which include various types of knowledge and intellectual skills; affective factors like attitudes, values, and motivations; personality traits, for instance, self-confidence; and social skills, encompassing abilities in communication and cooperation [23]. Ellström [21] suggests that competence should be seen as an interaction between an individual’s (or collective’s) potential capacity and the conditions, requirements, and opportunities presented by the specific job or task. The notion of competence-in-use [21, 24] captures the individual’s engagement with workplace affordances, as discussed by Billett [25]. In summary, Ellström’s perspective encourages a more dynamic, contextual, and holistic approach to understanding and developing competence in the face of organisational change.

The ensuing sections of the paper will present the methodology, followed by the analysis and discussion of our findings.

## 2 Method

To answer the study’s purpose, an integrative literature review was conducted [26, 27]. This method provides advantages in terms of describing emergent areas of research based on empirical studies with both quantitative and qualitative data [28]. As a first step, focus, content, and limitations were established based on the review’s purpose. Next, inclusion criteria were formulated for studies to include or exclude in the search and review process.

The inclusion criteria were as follows: a) workplaces/organizations and planned organizational changes; b) the ability to apply existing competence, the ability to develop new change competence; c) individual well-being, productivity, learning and innovation. The studies were also required to be d) scientific articles published in international, peer-reviewed (academic) journals; e) written in English, Swedish, Norwegian, or Danish; and f) contain empirical material. No specific time frame was set for the articles to be included in the study. Hence, all articles published until 2023 were considered. The inclusion and exclusion criteria were then established, and a list of studies that met the criteria was created. This list consisted of various studies, some of which were not empirical, and were already known to the authors. These studies were analyzed to generate relevant search terms and to validate the initial searches. Based on the results, the search terms were further refined and used for subsequent searches.

**Fig. 1:**
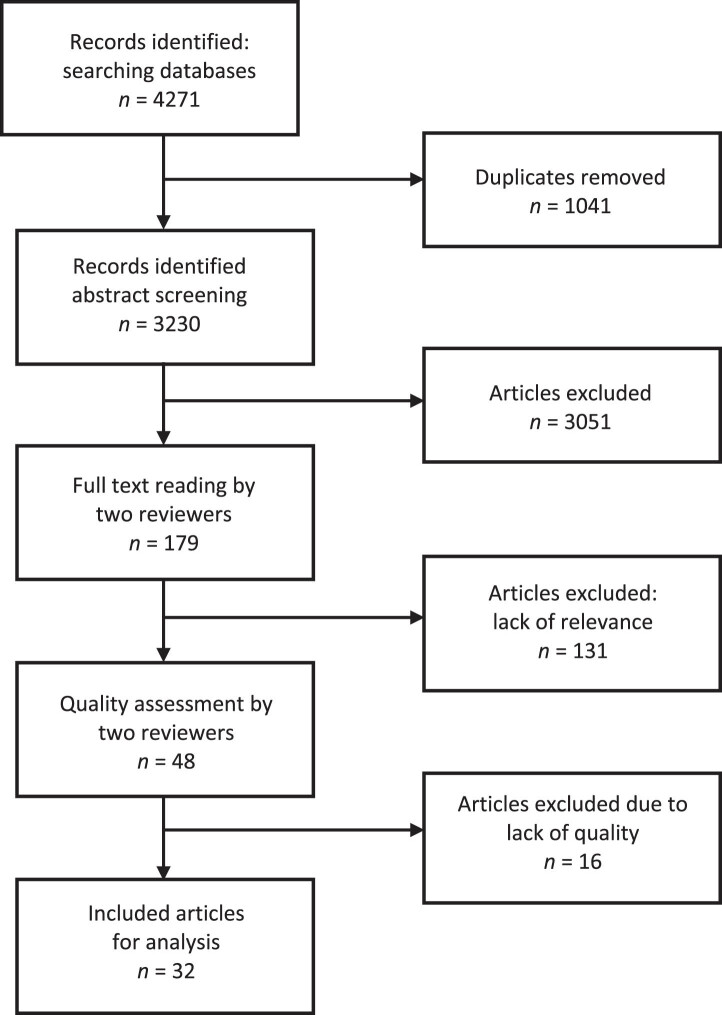
Flowchart over the article selection process. (Scopus: 2634, WoS: 1637).

The searches were conducted in Scopus and Web of Science and produced 3230 unique studies (Fig.  1). Examples of search terms used to capture organizational change and change competence were: “Organizational changes” or “Intended change” or “Change interventions” or “Corporate change” or “Reorganizing workplaces” AND “Change competence” or “Change capacity” or “Change agency” or “Organizational performance” or “Change acceptance”. The search was conducted in January 2024.

The studies were imported into Rayyan software with the function “blind” on. The studies were screened by two of the authors based on the title and abstract. Next, the blind function was unlocked, and the studies that had been marked differently were all read through and discussed until a consensus was reached. In total, 179 were selected for relevance review based on the inclusion criteria for the literature review. After reviewing the downloaded full texts for relevance, 48 studies met all inclusion criteria. These 48 studies underwent a quality assessment based on recognised protocols for quantitative [29, 30] and qualitative studies [31]. All studies were reviewed and graded in a three-point quality assessment: low, medium-high, or high quality. Of the 48 quality-reviewed studies, 32 were assessed to be of high or medium quality, of which 14 were quantitative, 15 were qualitative, and three involved multiple methods. Thus, a total of 32 studies were included in the review.

The qualitative studies were assessed in several steps. After an initial reading, basic information about the paper, such as journal, country, purpose, etc., was collected, and an overview of the articles emerged. The second reading was focused on results, discussion, and conclusions as a basis for a conventional inductive content analysis [32], and preliminary categories were created from each article. In this step, each article was summarised with a focus on the content and validity of the findings. The studies were also described in tabular form regarding purpose, method, and results. In the third and final step, the preliminary factors identified in step two were discussed, giving the overall categories for organizational change competence.

In the analysis process of the quantitative studies, all studies were read, and tables with key information relevant to this literature review were compiled. This is called a narrative synthesis [33]. Such key information included method and design, population, year and country for data collection, organizational change competence measures, bivariate associations, and final models (with possible consideration of other factors). In the third and final step, key findings for each study were compiled and then inductively clustered into four provisional groups, ultimately resulting in two main themes with two subgroups each:

1. Process (how change is carried out)
2. Conditions (for organizational change)

At least two authors performed each step of the literature review to increase reliability.

This study, as a literature review, is exempt from Institutional Review Board approval.

## 3 Results

This literature review incorporates 32 studies published between 2002 and 2022, all appearing in various scholarly journals (see Table 1). However, The Journal of Applied Behavioral Science was a common publication source, hosting four of these 32 studies. The selected studies’ methodologies varied: 14 employed quantitative methods, 15 utilised qualitative methods, and three incorporated a mix of both. 18 studies were cross-sectional studies and used 14 longitudinal data. Regarding the sectors examined by these studies, the majority (21 out of 32) explored empirical data from public sector organizations, primarily focusing on healthcare— conversely, nine studies centred on private sector data. Two studies evaluated empirical data from organizations operating in both the public and private sectors. Additionally, an aspect worth noticing is that none of the studies had an explicit critical perspective of organizational change, such a analyses of power dynamics or gender perspective.

**Table 1:** Overview of included studies

Author(s) and year	Country and organization	Change	Focus	Methods and samples
Alänge &Stelber 2009	Sweden 2 industrial companies, 1 hospital	Implementation of a new quality system (hospital), new production system (industrial companies)	The board’s importance for sustainable organizational changes	Case studies 18 interviews with CEOs, board members (key persons)
Augustsson et al. 2017	Sweden 78 primary health care centers	Implementation of a new IT- and communication system, competence investment	Attitudes to change at individual and group level, based on change content and process	Longitudinal study Questionnaires (*n* = 1042, 41%), all staff categories
Balogun 2003	U.K. Hospital	Privatization of a hospital. Change of structure, system, work methods, and organizational culture	Middle managers’ role in a major organizational change	Case study 26 interviews with middle managers and diaries in 3 units
Balogun &Johnson 2005	U.K. Hospital	Privatization of a hospital. Change of structure, system, work methods, and organizational culture	Middle managers’ sensemaking during organizational changes	Case study 26 interviews with middle managers and diaries in 3 units
Bartunek et al. 2006	USA Hospital	Changes in nurses work tasks with more responsibility, more opportunities to influence and control work, and to participate in decision processes	How change initiatives are received by the recipients	Cross-sectional study Questionnaires (*n* = 501, 48%), archival data.
Bordia et al. 2011	Philippines University	Merger of academic units and implementation of a new educational facility	How poor change leadership can affect employees’ attitudes and actual turnover, and tests a model	Longitudinal study Questionnaires (*n* = 124, 62%) to employees in various academic departments
Clark et al. 2010	USA 2 healthcare organizations	Merger of two healthcare organizations	The importance of organizational identity in successful organizational change	Longitudinal case studies 33 interviews, observations, document studies
Darling et al. 2021	Canada Hospital	Implementation of a midwifery unit in a hospital	How inner and outer characteristics, individuals, and processes influenced the implementation of a midwifery unit	Case study 15 interviews with managers, obstetricians, midwifes and one midwife consumer, document analysis
Eilers et al. 2022	Switzerland Organizations in different sectors	Managers’ and trainers’ use of agile mindset to handle changes	How actors’ agile mindset can predict strategic agility and performance	Cross-sectional, mixed method 15 interviews with senior managers, agile coaches, trainers, developers. Questionnaires (*n* = 449) to participants in agile experienced organizations (test of model)
Griffin et al. 2003	Australia Public organization	Employees’ change activities in the organization; different sources of change, supportive leadership, staff enthusiasm	Whether organizational change has different effects on group leadership and morale depending on who (at what level) initiates the change	Longitudinal study Questionnaires (t1 *n* = 3355, 73%, t2 *n* = 3314, 72%), various staff categories
Hetzner et al. 2009	Germany Bank	Implementation of a new standardized method for customer advice for bank advisers	How employees perceive planned change and learning opportunities	Case study 10 interviews with client advisors
Hornung &Rousseau 2007	USA 2 hospitals	Shared leadership intervention: decentralizing decision-making, creating a more supportive work environment	The importance of autonomy for employees’ productivity and the effects on employees’ support for change	Longitudinal study Questionnaires (t1 *n* = 166, 47% and t2 *n* = 74 18%), various staff categories
Huy 2002	USA Units in a major IT company	Divisionalization of a company, new management team and CEO	How middle managers can contribute to employees maintaining an emotional balance during radical change	Longitudinal case study 265 interviews with various staff categories (most upper and middle management)
Jimmieson et al. 2008	Australia Local government	A building relocation, the first phase	How attitudes, norms, individual behavior, and the interactive effect of group norms and identity predict willingness to participate in change	Cross-sectional study Questionnaires (*n* = 151, 84%), various staff categories in 18 departments
Jones &van de Ven 2016	USA 15 hospital clinics, 40 primary care clinics	A merger of 55 clinics, an integration of new structures, management practices, systems, and climate	The relationship between change resistance, its consequences, and how potential causes are strengthened or weakened over time	Longitudinal study Questionnaires (*n* = 289, 33%), various staff categories
Karlsson &Nordström 2022	Sweden Hospital	Implementation of a hospital-based home rehabilitation program	How use and exchange of knowledge can facilitate change work and methods to support managers	Case study 7 individual interviews with project members and managers, 1 group interview with the rehabilitation team
Kavanagh &Ashkanasy 2006	Australia 3 universities	A merger of several divisions and departments at three universities	The importance of leadership and change strategies for organizational culture and change acceptance	Longitudinal study, triangulation Questionnaires (t1: *n* = 203, 38%, t2: *n* = 152, 73%, t3: *n* = 123, 81% of 152) and 3 (t1) and 60 interviews (t2) with employees
Kim et al. 2011	USA Hospital	Implementation of a change program, including shared leadership, in a hospital	Conditions or factors that can lead to change-supporting behavior and how they can vary during a change	Longitudinal study Questionnaires (t1 *n* = 166, 47% and t2 *n* = 207 52%), various staff categories
Koivisto et al. 2013	Finland Public organization	A move of a public organization (ordered by the government) to another town	The significance of whether the organization and leaders are perceived as fair during change	Cross-sectional study Questionnaires (*n* = 104, 54%), all employees
Lee &Choi 2021	Korea Manufacturing industry Small Medium Enterprises (SMEs)	Implementation of green supply chain in different SMEs	The relation between partnership governance mechanism (suppliers and manufacturers) and green supply chain management	Cross-sectional study Questionnaires (*n* = 202, 24%) to supply chain managers in various organizations
Lines 2005	Norway Bank, insurance, ship building, furniture	Managers participating in a management program for strategy development, and their experiences of change processes	How different types of organizational changes can lead to organizational learning	Cross-sectional study Questionnaires (*n* = 88, 48%) to middle managers participating in a leadership program
Neves 2011	Portugal 2 public organizations, University, city hall	Implementation of a new performance appraisal system at a university and implementation of flexible work schedules in a city hall	How commitment to change develops and the importance of competence and support from leaders and management	Cross-sectional study Questionnaires to all employees in 2 public organizations (*n* = 122, 77% and *n* = 88, 77%)
Rowland et al. 2018	Canada Different units in healthcare	Planned change within healthcare to increase patient participation	To increase patient participation	Case study 26 interviews with staff and patients from various units
Rubin et al. 2009	USA 7 industrial plants	Investigates leader cynicism during organizational change	How leaders’ attitude towards change affects employees’ attitudes	Cross-sectional study Questionnaires to managers (*n* = 106, 80%) and their employees (*n* = 933, 100%)
Stensåker et al. 2008	Norway 3 units in a major company	Streamline structures, work processes, leadership, control system, implementation of a new IT system	How the implementation of planned changes can affect sensemaking among employees	Longitudinal case study 84 interviews with various staff categories, 39 observations, and document analyses
Stensåker &Myers 2012	Scandinavia 10 companies	Top- and middle managers’ experiences of large-scale changes in their companies	The importance of previous experience with changes and how employees react to organizational change	Case study 50 interviews with various staff categories
Szabla et al. 2014	USA Graduate school	Change recipients’ reflections on a recent organizational change, and perceptions of change strategies	Managers’ competence in selecting the correct strategy depending on the type/nature of the change	Cross-sectional study Questionnaires (*n* = 88, 33%), PhD students and PhDs
Vallealla et al. 2015	Finland Hospital	Emergency nurses were given more/new duties, more responsibility and authority, and a fast track was introduced for the treatment of minor ailments	Learning opportunities in a planned change	Longitudinal case study 16 interviews with nurses, meeting observations
Van der Voet 2016	Netherlands Municipal departments	Implementation of a reform program to restructure administrative departments and centralize their back offices	The relation between immediate supervisor’s change leadership and employee willingness/commitment prior to change	Cross-sectional study Questionnaires (*n* = 515, 364%), employees in 2 entities
Westerberg &Tafvelin 2015	Sweden Home care service	Managers’ importance for creating/increasing commitment among employees in homecare	Leaders’ importance for commitment to change	Longitudinal study 2 rounds of interviews with 10 supervisors and 8 middle managers
Wylie &Study 2018	U.K. 24 organizations, public/private	Change agents’ role/assignment during change	Identifies, describes, and assesses various types of collective change agents	Case studies 96 interviews with change agents

### 3.1 Process (how change is carried out)

The first theme concerns two sub-themes: 1) leading and managing change, mainly from the perspective of managers and 2) employees’ participation in decision-making during change.

#### 3.1.1 Leading and managing change

Fifteen studies concern managers and management, but also trainers and change agents and their mission to lead and manage change. Managers’ ability and different strategies to lead change were investigated in four of the studies [34–37]. Kavanagh and Ashkanasy [35] found that important leadership skills during change were sympathy towards the employees, communication and transparency about the change process. The study points out the importance of leaders’ ability to lead a change process and that changes forced upon the leader take time to incorporate into the organizational culture.

Eilers et al. [34] investigated the agile mindset among managers, trainers, and developers in different organizations and sectors. A relationship was found between an agile mindset (comprising learning spirit, collaborative exchange, empowered self-guidance, and customer co-creation) and performance mediated by strategic agility.

Lee and Choi [36] investigated how small and medium-sized enterprises complied with green initiatives and found that transactional mechanisms between suppliers and organizations were associated with green supply chain management performance. The study implied that managers must assess and develop their relationship with partners as a successful green innovation implementation requires a relation governance mechanism, which also contributes to organizational performance. Wylie and Sturdy [37] examined internal collective change agencies and identified four different kinds of collective change agencies: transformers, specialists, enforcers, and independents. Transformers were to deliver major changes across the entire organization, often temporary and independent of other organizational entities. Specialists were responsible for more limited changes, had more embedment (e.g., IT and HR), and were often experts in their respective fields. Enforcers focused on the entire organization with a high degree of embedment in the organizational structure. Due to their proximity to the senior management, enforcers were often met with suspicion. Independents were operators for limited tasks within limited parts of the organization. By identifying groups of collective change agencies, the authors claimed that it is possible to see a shift concerning the meaning of change agency in the direction of more collective forms.

Five studies [38–42] investigated the importance of managers’ ability to translate or interpret the change process and how recipients receive change. Balogun [38] examined the importance of middle managers in a comprehensive change process that involved changes in structures, systems, working methods and organizational culture. The study showed that managers’ activities involve interpreting, understanding, and translating different meanings of change between various parties. Similar results were found in the study by Szabla et al. [42], who found that managers used different change strategies depending on the type of change. The authors also identified several factors that influenced leaders’ strategies. The study showed that the ability to interpret, understand and decide what strategy to adopt is thus a key competence for a leader.

The study by Bartunek et al. [39] examined how change was received by individuals assigned to implement change that was initiated by others. The results showed that change was perceived as positive when information about the change and its significance for their work was consistent. The study showed the importance for leaders or change agents to provide a holistic picture of the change and how it will be implemented to increase understanding and insights regarding the advantages and disadvantages that the change may bring. Griffin et al. [40] studied differences in group attitudes towards leadership and morale during organizational. The results showed differences regarding how change was received, depending on which hierarchical level initiated the change. Changes initiated by a supervisor or a line manager received more support and positive responses than changes initiated by higher managers and the management.

Lines [41] investigated the relationships between social accounts, participation, and organizational learning during change. The sample consisted of managers participating in a management program for strategy development. In the results, social accounts (social values, policies) and participation were found to have a direct positive relationship with organizational learning, but the influence of social accounts was negatively moderated by participation. A possible explanation is that increased participation can reduce the significance of social accounts in organizational learning, i.e., social accounts become less significant. According to Lines [41], this may indicate the importance of clarifying and working with the organization’s values and expertise, but also to involve employees during change to increase understanding of the change process.

The importance of managers acting supportive during change was investigated in four studies [43–46]. In van der Voet’s study [46], the relationship between change leadership and employee commitment to change was investigated. The results showed indirect associations via relevant communication and the level of participation, but also that support from the immediate supervisor was important.

Huy [44] found that due to managers’ awareness of employees’ emotional needs during change, operations could continue without disruption. This capacity came with experience as managers learned how to balance employees’ emotional needs in the tension between continuity and change. Although the study by Jones and van de Ven [45] focused on resistance, the results also addressed how organizations can handle resistance. The results showed that supportive leadership reduced change resistance, suggesting that leaders can work actively to support their employees throughout the change process and thereby mitigate the resistance. The study by Balogun and Johnson [43] questioned the common opinion of planned changes as rational and goal-based. The results showed that a major part of the sensemaking occurred among the managers horizontally without higher managers. Vertical sensemaking was often built on formal planning and goals associated with the change, while horizontal sensemaking was more informal and built on changes in the managers’ work. The study indicates that leading change is not about steering, managing, and controlling but rather about supporting and creating favourable conditions for a more collective sensemaking process concerning the change’s purpose, objectives and expected results.

Two studies [47, 48], investigated sensemaking and commitment from the board or top management. Alänge and Steiber [47] found in their study that commitment from the top management played a crucial role in the sustainability of the implemented change. The top management should possess sufficient experience and competence to support organizational change with an open, curious, and cooperation-based culture rather than focusing on control.

Clark et al. [48] studied a merger of two healthcare organizations with different organizational identities and the challenge to create meaning for their future partner. The two organizations had major problems revising or releasing their existing perceptions and organizational identity. This was solved by talking about the future organization as a temporary and non-binding construction with a temporary name, which enabled the process to move forward. The picture of the future organization served as a transitional identity and a transitional object that could bridge the differences in perceptions between managers and employees in the two organizations.

#### 3.1.2 Employee participation in decision-making

Employees’ participation, autonomy and decision-making in change activities were investigated in four studies [49–52].

Hornung and Rousseau [50] examined how employees at two hospitals perceived their degree of autonomy, the importance of autonomy for proactivity and the decision to support change. The study showed that autonomy affected self-efficacy and personal initiative, which were associated with employees’ responses to organizational changes.

Mendy [51] investigated how small and medium enterprises (SMEs) confronted practical problems and challenges when implementing changes in their work practices and policies. When the management failed, employees identified spaces where they could position their preferences and handle obstacles and barriers, showing the importance of bottom-up recognition regarding difficulties, adversities, conflicts, and tensions when implementing change.

Stensåker et al. [52] found that participation in planned organizational changes fosters motivation and supports collective sensemaking. However, the authors highlighted the risk of seeing participation as a decisive factor for successful change. Participation in the planning phase is often insufficient, as problems often arise during the implementation of the change.

Darling et al. [49] followed the implementation of a midwifery unit and identified six essential conditions: 1) innovation; 2) the outer context; 3) the inner context; 4) communication and influence; 5) linkage between the design phase and the implementation stage, and 6) the implementation process. The results also showed that decision-making devolved to the frontline teams, leadership engagement, human resource issues, dedicated resources, internal communication, reinvention and development, and feedback on progress were associated with the success of the implementation process.

### 3.2 Conditions (for organizational change)

The second theme concerns conditions for carrying through organizational change in terms of attitudes and behaviour during change, as well as opportunities for competence exchange and learning.

#### 3.2.1 Attitudes to change and change behaviour

Six studies [53–58] investigate employees’ and leaders’ attitudes towards change.

Jimmieson et al. [55] found that employees’ attitudes, norms and perceived behavioural control predicted intentions to support organizational change. Group norms had an additional significance, where individuals who strongly identified with their group followed the group’s intentions to a higher degree. Information, communication, and participation opportunities indicated higher intentions to engage in organizational change activities. The focus in studies by Koivisto et al. [56] and Rubin et al. [57] was leader cynicism towards organizational changes and its negative impact on employees’ attitudes. The study by Koivisto et al. [56] showed that leaders with a cynical attitude towards change had a negative impact on employee attitudes and decreased their performance. A pessimistic attitude towards change was also found to be negative for the leader, as management and higher managers may score the leader’s performance and commitment as low. Rubin et al. [57] found that leader cynicism about organizational change affected both the leader and his/her employees negatively. The study showed that leaders who believed the change was positive were also more transformative in his/her leadership and more productive, which in turn had positive effects on the employees. The study by Westerberg and Tafvelin [58] showed that leaders could express both positive and negative opinions towards planned changes, but the opinions became more homogenous over time. The most committed leaders confronted several obstacles during change, while most leaders gradually came to accept and consent to the change. Reasons for the upcoming convergence in perceptions were also associated with managerial training and regular meetings with the management during the change process. The study concluded that attitudes towards change alter over time, and regular meetings between different organizational levels can contribute to consensus. Augustsson et al. [53] and Bordia et al. [54] investigated openness to change. The study by Augustsson et al. [53] aimed to investigate how openness to change, in relation to change content and change process, affected daily practice and the use of acquired information and communication technology (ICT) competence. The results at the individual level showed that openness to the change process predicted higher levels of ICT competence and higher use of ICT competence. Openness to change content predicted higher levels of using acquired ICT competence but not higher ICT competence. At the workgroup level, openness to change content predicted a higher level of using acquired ICT competence and higher ICT competence. The study indicates that openness to change is an important predictor of successful outcomes for the individual as well as the workgroup. Bordia et al. [54] examined the effects of Poor Change Management History (PCMH) on job satisfaction, trust, willingness to change jobs and openness to change and how that may affect actual turnover. The results of the study showed that previous experiences of poorly implemented changes in the organization led to lower trust, decreased job satisfaction and openness to change, and higher cynicism and turnover intentions.

Two studies investigated change behaviour [59, 60]. In the study by Kim et al. [59], conditions regarding positive change behaviour, i.e., actively participating in, facilitating, and contributing to change, were investigated. The results showed that anticipated benefits of the change and the quality of the employment relationship were positively related to change-supportive behaviour but moderated over time. While anticipated benefits became less important over time, employment relationship quality became more important for predicting change-supportive behaviour. The study showed that different conditions are of different importance over time during a change process, and employers must actively support their employees and get them involved. The study by Stensåker and Meyer [60] focused on employees’ previous experiences of major organizational changes and their significance for employee reactions during ongoing organizational changes. The results showed that positive experiences contribute to greater solidarity, while negative process-related experiences lead to lower solidarity (even cynicism). Managers and supervisors play an essential role in developing change capability among employees.

#### 3.2.2 Opportunities for exchange and learning

Five studies investigated the exchange of experiences, knowledge, competence, and learning opportunities during change [61–65].

Karlsson and Nordström [62] investigated how the use and exchange of knowledge can facilitate change, support managers, and improve healthcare quality. The results showed that exchanging knowledge, collaboration and learning across boundaries could facilitate change in healthcare and that communication must be planned and prioritized.

Rowland et al. [64] studied planned changes in the healthcare system to increase patient engagement. The results showed that patients’ experiences contributed to a better understanding of how healthcare services can be designed, delivered, and implemented. The authors argue that organizations may not learn from patients per se but learn from patients’ subjectivity by allowing patients’ experiences to impact ongoing organizational changes.

Hetzner et al. [61] studied bank advisers’ perceptions of planned changes, requirements for learning, and factors supporting or inhibiting learning in the context of change. The results of the study showed that although employees perceived that work tasks became more routine-based, (time-) intensive, and complex, the change did entail opportunities to develop new knowledge and competence as well as more significant between the advisers.

Neves [63] found in his study that employees who perceived their manager as competent also experienced greater support from the manager. Managers’ perceived competence was also found to be negatively associated with long-term or continuous commitment to change. A possible explanation, according to Neves [63], is that leaders’ competence may decrease employees’ fear of change to a higher degree than support. The study showed that leaders considered highly competent and supportive during change enhance employee commitment to the change.

**Fig. 2:**
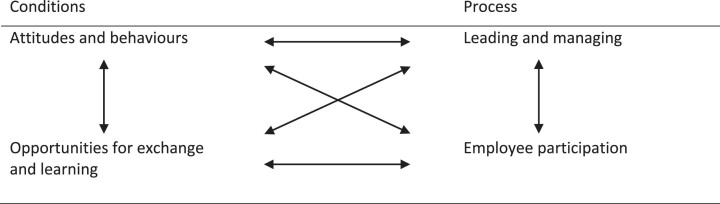
Conditions and process relations.

Valleala et al. [65] focused on employee learning opportunities in an emergency clinic during an organizational change. Experienced nurses were promoted as supervisors during their shifts, which included new job duties, responsibilities, and authorisation. Although the nurses could not attend all meetings and most of the critical decisions were taken by managers, participating in parts of the change process increased the nurses’ opportunities for organizational learning, where they could discuss problems and solutions. The learning opportunities at the organizational level were also expanded, as work methods and changed organizational roles changed.

## 4 Discussion

This integrative literature review has studied research investigating competence during an organizational change process. The analysis of the 32 included studies resulted in two main themes, ‘Process’ and ‘Conditions’, with two sub-themes each. The sub-themes belonging to ‘Process’ concerned leading and managing change processes and the importance of employee participation and decision-making in organizational change processes. The sub-themes belonging to ‘Conditions’ dealt with employees’ attitudes to change and change behaviour, competence exchange and learning opportunities during change.

### 4.1 Four themes of competence during change processes

Leading and managing change processes mainly concerned the necessity of managers and change agents adapting their leadership to different situations and upcoming demands during change for a successful change process, but also that their competence was a source of legitimacy during the change. A good match between demonstrated leadership and situational requirements increased employees’ trust in the change and was based on mutual communication [35, 38, 42, 44, 45, 63].

Employee participation in decision-making was primarily about employees affected by the change and their need to feel involved. This does not necessarily mean altering the planned change, but they need to feel included, listened to and allowed to express criticism or dissatisfaction [49, 50].

Attitudes and behaviours mainly concern the attitude towards change and its importance for the implementation and success of the change, including previous change experiences and pressure from colleagues and peers. In this sub-theme, the focus was primarily on employees [53–55, 59]. However, managers were also mentioned as role models during change [56–58].

The sub-theme Opportunities for exchange and learning emphasized experience- and knowledge exchange, but also further distribution, as vital aspects of a successful change. Collaborations within and outside the organization, were highlighted as they led to increased competence that could be used in the change [61, 62, 64, 65].

Although the two main themes and four sub-themes describe essential characteristics of competence during organizational change, they also seem to be related. The existing conditions affect the process, i.e., how change is implemented. At the same time, the process creates new conditions for continued change. The study’s themes and their relationships are illustrated in Fig.  2.

The two sub-themes concerning conditions for change are related in that they may reinforce each other. A negative attitude towards change among individuals and work groups reduces the willingness to share competence. In contrast, a positive attitude towards change increases the desire to learn and share competence (e.g. 53). Similarly, sharing knowledge and experience may influence the attitude toward the change.

The two sub-themes involving the process of change are also related. There is a tension between leading and managing towards the decisions and goals the change implies, and on the other hand, allowing employee influence and involvement and thus opening for alterations of the change [43, 46]. This tension highlights the power dynamics within the organization. Planned changes are often initiated and controlled top-down. Most resources, in terms of time and budget, are spent on planning and decision-making at higher organizational levels, while less resources are spent on implementing the change and involvement of all hierarchical levels. Employees are often involved in the implementation phase when all decisions have been made. This may undermine a sense of involvement, and dissatisfaction or questioning often are silenced instead of highlighted. Thus, the tension in the process also influences the conditions, in terms of attitudes and behaviour. If employees affected by a change do not feel included, the risk of dissatisfaction, resistance and a negative attitude towards the continued change process increases. An inclusive process may improve the possibility that employees perceive the value of the change and contribute with constructive behaviours [43]. At the same time, previous experiences of changes may affect the attitude regarding participation [60], and negative previous experiences can depress participation if no value is found (window dressing, sham democracy). Thus, the conditions influence the process.

### 4.2 Competence during change processes at different organizational levels

Based on the results of the included studies and the identified themes with their interrelationships, it becomes clear that organizational change, its conditions, and its process differ at different levels in the organization. With that, the competence needed also differs during an organizational change. In organizational research, there is a long tradition of dividing the organization into functions or levels based on the degree of operational or strategic activities performed. Applying a competence perspective to different levels clarifies how the required competence during change varies depending on the level that is in focus.

The operative part of an organization consists of individual employees. They possess specific professional competence and experience, and through their professional competence, employees have the best opportunities to see potential challenges and problems during changes in operational activities. During change, employees need to adapt their existing professional competence to reach the goal of the change. Change competence at this level consists partly of identifying and conveying difficulties with the intended change and partly in adapting existing practices following the intention of the change [51, 61, 62].

A level higher, but still focused on performing the operational activities, is the workgroup and the workplace. This level constitutes the social context in which an organizational change is implemented and where employees often seek support and help. At the same time, it is important to note that this social context can also inhibit individuals and for the change process through various social mechanisms such as social conformity, group pressure or groupthink. Change competence at this level consists of finding a common translation and interpretation of the organizational change and a common strategy that contributes to the intended change [52, 53].

The change agent constitutes the next level which can be considered as an intermediate level with operational and strategic tasks during a change process. A change agent is the individual or the individuals responsible for implementing the change. A change agent can be a manager or temporary project leader. This role constitutes a central link and mediator during a change process between executors and decision-makers (which includes communicating in both directions). Change competence at this level mainly consists of being able to read and act in different situations, communicate, inform, push, support, listen or keep quiet, and push the change process forward [34, 39, 40, 42].

The final and highest level that can be identified in the findings of this review is the top management. This level is the most strategic, where most decisions about planned changes are made. However, it is essential that this level initiates the change process and is involved throughout the change. Change competence at this level mainly consists of continuously informing about the change process and its overall goals and visions during the change process, being involved in the change process, and showing clear support for the change agent. The top management must also be receptive to new ideas and revisions based on feedback given from the operational levels during the change process. This provides confidence to the change in the organization and gives legitimacy to the change agent [47, 48].

Change competence can thus be found at different levels in an organization, but with different content depending on whether it concerns an operational or strategic role in the change process. Based on Ellström’s [21] reasoning and the findings of this review, the meaning of change competence can be described as the potential capacity to utilise existing competence within the organization as well as the ability to use and develop competence to be able to meet external or internal generated needs for organizational change. It entails questioning and testing established or assumed tasks, goals, and conditions related to professional roles, quality, productivity, and working conditions. It requires individuals or collectives to surpass the given or taken-for-granted boundaries encountered in their activities, demonstrating a proactive and adaptive approach to navigate and shape change within the organization. Sharing competence and experiences during a change process may thus develop into learning. From an organizational perspective, an organization’s change competence can thus be understood as organization’s ability to use existing competence and to develop competence to deal with internal needs of change and to develop resilience to external change pressures.

### 4.3 Implications

Organizational change is a research area that has gained much attention for several decades. The theoretical contribution of this literature review is the concept of change competence, which, in contrast to other nearby concepts capturing planned changes, includes exchanging knowledge and learning opportunities during organizational change. In this way, previous concepts are brought together in one overarching conceptual framework that can be used in future research to further the understanding of necessary prerequisites during organizational changes.

This review also has practical implications. The findings highlight that managers and change agents are significant during change, not only for managing and leading change but also for supporting and visualizing competence and experiences that exist among all in the organization. It is also essential that managers inform and involve all parties concerned early in the change process and make sure there are actual opportunities to participate (e.g., time). The change needs to be framed so it is clear what it implies, why it is implemented, what the change is expected to lead to, how all parties may be affected by the change and their role. In this way, good attitudes can be created which are significant for learning. Expressed dissatisfaction, criticism, or questioning should not be silenced. Instead, take advantage of opinions, comments, and experiences to enrich and adjust the change. Learn from the past by evaluating the results of the change and use previous experiences, both successful and less successful. Finally, much of the resources are placed on planning the change with great faith in the creation of policies and goals rather than focusing on the actual implementation. Perhaps the investment of resources should be the reverse –more resources on development and support when implementing the change. There must also be a balance between implementing the change and ordinary production and assignments.

### 4.4 Limitations

This literature review acknowledges certain limitations. The body of literature on organizational change and workplace competence is extensive. A variety of search terms were utilized to ensure the inclusion of relevant studies. Despite this, some relevant papers may have been missed if certain terms were overlooked.

In discussing the themes emerging from the review, several significant tensions were identified that merit further investigation. While the primary goal of this paper was to elucidate the concept of ‘change competence’, delving into other theoretical perspectives was considered outside the scope of this study. For example, examining competence during organizational change through a lens of power dynamics or focusing on the gender structure within organizations could provide additional insights. Still, this paper did not pursue such analyses due to the focus on change competence.

## 5 Conclusions

This integrative literature review sets out to explore the concept of ‘competence’ in the context of organizational change processes, and what this concept may entail at different organizational levels. The findings suggest that competence is a unifying concept that can be considered both as a central condition in change processes and as an important aspect during the change process itself. Competence during a change process includes attitudes to the change and opportunities to exchange experiences, but competence also includes management and participation in the change process.

The findings also suggest that change competence can be found at all levels within an organization but with different content depending on the mission or role in the organization and the change process. Change competence for employees and work groups comprises identifying problems with intentional change but also enabling work practices in accordance with the change. Change competence for change leaders and managers comprises informing and convincing those involved in the change, but also adapting the change based on identified problems. An organization’s ability to meet future rapid transitions may thus depend on its ability to apply existing competence, and through learning, develop change competence.

## 6 Ethical approval

Not applicable.

## 7 Informed consent

Not applicable.

## Conflict of interest

The authors declare no conflict of interest.
